# Toxicity on Social Media During the 2022 Mpox Public Health Emergency: Quantitative Study of Topical and Network Dynamics

**DOI:** 10.2196/52997

**Published:** 2024-12-12

**Authors:** Lizhou Fan, Lingyao Li, Libby Hemphill

**Affiliations:** 1 School of Information University of Michigan Ann Arbor, MI United States

**Keywords:** social media, network analysis, pandemic risk, health care analytics, infodemiology, infoveillance, health communication, mpox

## Abstract

**Background:**

Toxicity on social media, encompassing behaviors such as harassment, bullying, hate speech, and the dissemination of misinformation, has become a pressing social concern in the digital age. Its prevalence intensifies during periods of social crises and unrest, eroding a sense of safety and community. Such toxic environments can adversely impact the mental well-being of those exposed and further deepen societal divisions and polarization. The 2022 mpox outbreak, initially called “monkeypox” but later renamed to reduce stigma and address societal concerns, provides a relevant context for this issue.

**Objective:**

In this study, we conducted a comprehensive analysis of the toxic online discourse surrounding the 2022 mpox outbreak. We aimed to dissect its origins, characterize its nature and content, trace its dissemination patterns, and assess its broader societal implications, with the goal of providing insights that can inform strategies to mitigate such toxicity in future crises.

**Methods:**

We collected >1.6 million unique tweets and analyzed them with 5 dimensions: context, extent, content, speaker, and intent. Using topic modeling based on bidirectional encoder representations from transformers and social network community clustering, we delineated the toxic dynamics on Twitter.

**Results:**

By categorizing topics, we identified 5 high-level categories in the toxic online discourse on Twitter, including disease (20,281/43,521, 46.6%), health policy and health care (8400/43,521, 19.3%), homophobia (10,402/43,521, 23.9%), politics (2611/43,521, 6%), and racism (1784/43,521, 4.1%). Across these categories, users displayed negativity or controversial views on the mpox outbreak, highlighting the escalating political tensions and the weaponization of stigma during this infodemic. Through the toxicity diffusion networks of mentions (17,437 vertices with 3628 clusters), retweets (59,749 vertices with 3015 clusters), and the top users with the highest in-degree centrality, we found that retweets of toxic content were widespread, while influential users rarely engaged with or countered this toxicity through retweets.

**Conclusions:**

Our study introduces a comprehensive workflow that combines topical and network analyses to decode emerging social issues during crises. By tracking topical dynamics, we can track the changing popularity of toxic content on the internet, providing a better understanding of societal challenges. Network dynamics highlight key social media influencers and their intentions, suggesting that engaging with these central figures in toxic discourse can improve crisis communication and guide policy making.

## Introduction

### Background

The 2022 mpox outbreak was first reported in several countries in Europe and quickly became a global health crisis [1]. Mpox is a viral illness that can be transmitted from animals to humans or between humans through contact with blood or bodily fluids [2]. This disease was originally named “monkeypox” and was later renamed to reduce stigma and other issues during the 2022 outbreak [3]. This public health emergency was characterized by a large number of cases and a high rate of transmission, which posed a significant threat to public health globally. Health authorities and public health organizations were quick to respond to the outbreak, implementing measures to control the spread of the disease, providing health care and support to those affected, and making vaccination efforts to protect those who were not yet infected [4-6].

During the mpox public health emergency, social media platforms were used for public health communication and information sharing about the disease, its spread, and people’s feelings about it. This led to both positive and negative consequences, with accurate information being shared alongside misinformation and toxic comments [7]. Toxicity on social media is prevalent during health crises, with many individuals spreading misinformation, fear, and hate [8,9]. This can undermine public health communication efforts and create confusion and fear among the public. In addition, toxicity on social media disproportionately impacts communities considered historically marginalized, exacerbating existing health disparities and making it difficult for these communities to access accurate and trustworthy information during a crisis. Homophobia and racism were common in mpox discussions [7].

Understanding the online toxic discourse during the 2022 mpox public health emergency is crucial for several reasons. First and foremost, studying toxicity in mpox discussions helps identify the factors contributing to the spread of misinformation, fear, and panic, which can exacerbate the public’s response to the outbreak. Second, by analyzing the motives and patterns behind such toxic behavior, public health officials and researchers can develop effective communication strategies to counteract negativity and promote accurate information. Finally, understanding the prevalence and impact of toxic discourse allows us to explore the broader implications of online behavior on societal discourse and public opinion formation during health crises. To achieve this understanding, we chronicled original tweets, the online posts on the Twitter (Twitter, Inc) social media platform, from May to October 2022. We then used the Perspective application programming interface (API) [9,10] to identify *toxic tweets*, which are the rude, disrespectful, or unreasonable comments on Twitter that are likely to encourage individuals to leave conversations. We analyzed the toxic tweets to answer the following research questions:

- Aboutness—What are the toxic tweets about and how do they change through time?
- Diffusion—How do toxic tweets spread through online social networks?

The *aboutness* of toxic tweets highlights the connection between the evolution of toxic discourse topics and the motivations of the people posting these tweets. This analysis helps us understand the thoughts and concerns of the ongoing public health emergency for a significant portion of the public, including 330 million monthly users who are active on Twitter’s social network [11]. The *diffusion* of *toxic tweets* is summarized as the retweets network and mentions network. The retweets network can inform who initiated or distributed toxic comments, while the mentions network shows who was frequently mentioned by other users and thus should be aware of toxicity dissemination. In particular, we followed the analytical framework of 5 dimensions, namely, *context*, *extent*, *content*, *speaker*, and *intent*. The 5 dimensions in characterizing toxic discourse, as adapted from the Rabat Plan of Action [12], are crucial for understanding the relationship between toxicity on social media and public health policy.

Our study aimed to unravel the complex dynamics of toxicity on social media during the 2022 mpox public health emergency using advanced computational techniques. Specifically, we aimed to identify the thematic structures, the *aboutness*, and the network behaviors, the *diffusion*, that perpetuate toxic discourse to understand how such narratives spread and the role of influential network actors in this process. Ultimately, our study sought to offer actionable insights that can help design effective interventions to mitigate toxicity on social media in future public health emergencies and other crisis situations.

### Toxicity on Social Media

Toxicity on social media refers to rude, aggressive, and degrading attitudes and behaviors, which are exhibited in various forms, including harassment, bullying, or even the spread of hate speech and misinformation [13,14]. One of the primary causes of toxicity on social media is the anonymity and physical disconnect provided by the cyberspaces of online platforms. The online disinhibition effect magnifies the toxicity and facilitates the implementation of toxic ideas in daily life [15], which makes toxicity on social media a useful tool to anticipate extremes in public opinions and social dynamics. At the same time, toxicity on social media is easily contagious and can propagate quickly through social networks [16], where algorithms deployed by online social media platforms can contribute to the spread of toxic content by amplifying it and showing it to a larger audience [17].

Consequently, toxicity on social media can negatively impact the mental health and well-being of individuals, contributing to anxiety, depression, and low self-esteem, among other issues [18,19]. The aggravating toxicity on social media can exacerbate the culture of hate and division, causing harm to communities considered marginalized and making it difficult for racial and sexual minority individuals to engage in meaningful and productive online discourses and face-to-face activities [20].

Recent efforts in academia and industry aim to improve understanding of online toxicity and implement detection and moderation using socio-technical approaches. Researchers have gained insights into toxicity on social media from different perspectives. Guberman et al [21] quantified toxicity and verbal violence through crowdsourcing, which can be useful for the moderation of toxic contents. Almerekhi et al [22] and Lwin et al [23] identified triggers of toxicity on social media and provided insights into the causes of toxic discussions by analyzing topical and sentiment shifts in interactions. Wijesirivardene et al [19] found that meaningful context of online conversations can help highlight or exonerate purported toxicity.

Benefiting from increasingly less expensive cloud storage and computing, social media platforms have also started developing and deploying toxicity on social media moderation applications. Perspective API developed by the Counter Abuse Technology team of Jigsaw and Google provides free access to toxic content detectors that aim at enabling healthy conversations and reducing toxicity and abusive behavior [9,10]. Similarly, the OpenAI moderation end point is a tool for checking content’s compliance with OpenAI’s content policy, including the prohibition of the generation of hateful, harassing, or violent content [24].

In addition to analysis and moderation of toxic content, other research calls for public engagement to tackle toxicity on social media, including encouraging individual responsibility and positive behaviors, as well as raising awareness and education [25,26]. Despite efforts to study online toxicity, it remains challenging to fully eliminate the impact of existing toxicity and prevent the spread of new toxic content. It is still imminent and meaningful to keep track, enhance comprehension, and intensify awareness of toxicity on social media.

### Health Crisis Communications on Social Media

Health crisis communications on social media refer to the use of social media platforms to disseminate information and communicate during public health emergencies and crises [27]. Social media has become a vital tool for public health organizations and governments to communicate during crises and emergencies, as it can quickly and effectively reach a large, diverse audience, facilitate public engagement, and gather feedback [28,29]. Individual users of social media can also widely read others’ opinions about a health crisis, freely express their feelings, and receive timely feedback [30].

However, there are also several challenges associated with using social media for health crisis communications. One of the main challenges is health-related toxicity on social media, which can lead to confusion and fear among the public. For instance, during the COVID-19 pandemic, there was a surge in misinformation, conspiracy theories, and discriminatory remarks, which not only hindered effective public health responses but also fueled stigma, discrimination, and even violence against certain groups [31,32]. In addition, the volume of information and the number of sources on social media can be overwhelming for the public, making it difficult for them to discern what information is reliable and relevant [33]. Without proper planning and intervention, information on social media can negatively influence health crisis communications and even result in infodemics [34,35]. Thus, public health organizations and governments should develop clear and consistent communication strategies for monitoring and responding to social media and provide accurate and trustworthy information to the public.

There are several guidelines that are helpful for public health organizations and governments to refer to. The Center for Risk Communication suggests six best practices in public health risk and crisis communication: (1) accepting and involving stakeholders as legitimate partners; (2) listening to people; (3) being truthful, honest, frank, and open; (4) coordinating, collaborating, and partnering with other credible sources; (5) meeting the needs of the media; and (6) communicating clearly and with compassion [36]. Recent research on the COVID-19 pandemic also suggests providing relevant, accurate, and sensitive information to key public groups to minimize communication noise and guide desirable coordinated actions [33]. While these principles are carefully written, it remains challenging to implement them in practice, especially due to the complexity of social media’s role in health crisis communications.

## Methods

### Overview

**Figure 1:**
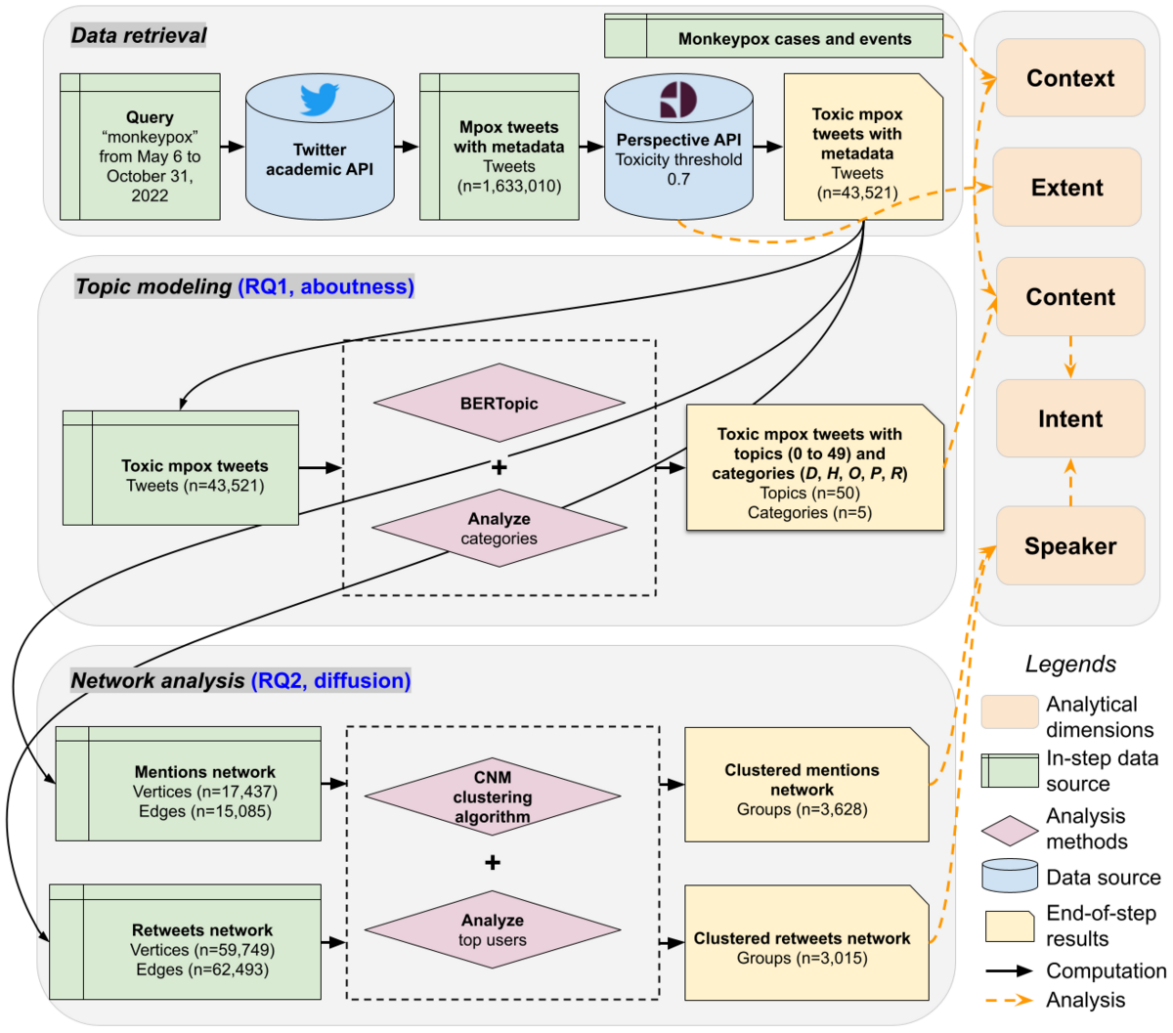
Workflow overview. We first used external application programming interfaces (APIs) to retrieve and identify toxic Twitter data and mpox context data. We then applied topic modeling and network analysis methods to categorize temporal topical dynamics and cluster network communities. We also mapped the results in each step to the analytical framework. BERTopic: bidirectional encoder representations from transformers–based topic modeling technique; CNM: Clauset, Newman, and Moore; RQ: research question.

We retrieved a large corpus of toxic online discourse on Twitter and applied computational methods for analysis. In this section, we describe the data and methods used in this paper (Figure 1). We first introduce the data retrieval process with *extent* as a content relevance filter, followed by a preliminary analysis of *context*. We then provide the details of methods for characterizing topical and network dynamics, supporting the comprehensive analysis of the *content*, *speaker*, and *intent* (of social media users).

### Ethical Considerations

This study uses publicly available Twitter data, strictly adhering to ethical guidelines and platform policies. All data were collected in compliance with Twitter’s terms of service, and efforts were made to anonymize any identifiable information to protect user privacy. While Twitter users consent to the platform’s terms, we recognize that this does not imply informed consent for research purposes; thus, the analysis of individual content was conducted with careful consideration to focus on public figures and avoid harm or misrepresentation. The research focuses on aggregate trends, ensuring that individual tweets are not decontextualized or used in a manner that could stigmatize individuals or groups. Finally, the findings are presented transparently, with an emphasis on societal benefit and respect for the original context of the data.

### Data

In this section, we demonstrate the data collection process and the context of volume peaks.

#### Retrieval of Toxic Tweets

To chronicle online discourse on the 2022 mpox public health emergency, we used the Twitter Academic API [37] to query tweets with the keyword “monkeypox.” Some relevant but less frequently used words, for example, “monkey pox” or “mpox,” were not included for query simplicity and API efficiency. As such, the name change did not influence our data collection. We archived a collection of 1,633,010 unique English-language tweets started on May 6, 2022, when the initial cluster of cases was found in the United Kingdom [38] and ended on October 31, 2022. This study solely analyzed publicly available and anonymized social media data. No identifiable information about individual users was collected, stored, or analyzed. All data processing and analysis strictly adhered to Twitter’s developer agreement and policy, ensuring compliance with ethical standards for privacy and confidentiality.

We then applied the Perspective API developed by the Counter Abuse Technology team of Jigsaw and Google to identify toxic tweets [9]. They define *toxicity* as a rude, disrespectful, or unreasonable comment that is likely to disengage others’ participation [9], especially those who are targeted by such toxicity. We adopted their definition of toxicity.

The Perspective API assigns each text submitted a probability score that corresponds to the proportion of people who would consider the text toxic. While choosing an appropriate threshold depends on the specific use case, the Perspective API team suggests that researchers experiment with a threshold between 0.7 and 0.9 to classify toxicity [9]. On the basis of our dataset, we observed that a tweet with a score ≥0.7 generally implied toxicity, and therefore, we chose 0.7 as the threshold to identify toxicity. When we limited our dataset to tweets that received a score of ≥0.7 from Perspective API, 43,521 toxic tweets remained.

#### Temporal Context and Hashtags

**Figure 2:**
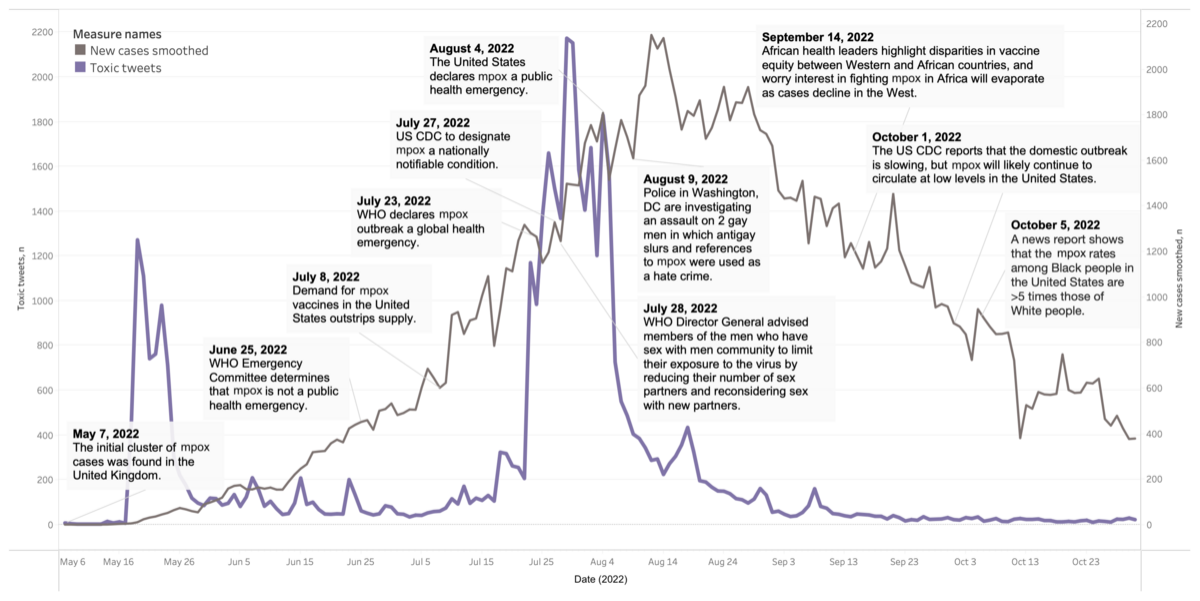
Development of the 2022 mpox outbreak [1] and the volume of toxic tweets. CDC: Centers for Disease Control and Prevention; WHO: World Health Organization.

As shown in Figure 2 [1], we observed 2 substantial peaks in the volume of toxic public discourse on Twitter related to the mpox public health emergency. The first peak occurred from mid to late May, with a daily high of >1200 toxic tweets. This spike in toxic discourse coincided with the reporting of the initial cases of the 2022 mpox outbreak [39]. Notably, this peak preceded the World Health Organization (WHO) public acknowledgment of their tracking efforts of the global disease development of mpox by approximately 1 month.

The second peak in toxic discourse was observed from late July to early August. This surge, which saw a daily high of approximately 2200 toxic tweets, nearly doubled the volume of the first peak. This increase followed 2 significant events: the WHO declared the mpox outbreak a global health emergency on July 23, 2022, and the Centers for Disease Control and Prevention designated mpox as a nationally notifiable condition on July 27, 2022 [1,2].

It is important to note that numerous mpox-related reports, particularly those with social and political implications, were released around and following the peak of new cases and the 2 peaks in toxic discourse volume. For example, on August 9, 2022, local police in Washington, DC, investigated an assault on 2 gay men as a hate crime; antigay rhetoric and references to mpox were used during the assault [40]. Then, on September 14 and October 5, 2022, there were reports on regional and racial disparities in health care, in particular, mpox vaccine equity, and health outcomes, that is, mpox rates [41,42]. These incidents underscore the profound real-world consequences of misinformation and toxic discourse, emphasizing the need for accurate and responsible communication on mpox and related issues.

Table 1 presents the summary of the top 20 hashtags in the toxic tweets, which highlights the frequent topics and provides a simplified overview of toxic online discourse on mpox. There is a diverse use of hashtags on different aspects of the mpox outbreak, including health emergency (eg, “pandemic” and “covid19”), health care (eg, “vaccine”), and population (eg, “lgbtq” and “gay”). These hashtags demonstrate the variety of topics in toxic tweets, which occur in different aspects of the online discourse.

**Table 1:** Top 20 hashtags used in toxic tweets (N=3742).

Hashtag	Count, n (%)
monkeypox	2889 (77.47)
monkeypoxvirus	106 (2.83)
covid19	102 (2.73)
covid	96 (2.57)
texasschoolmassacre	77 (2.06)
gay	76 (2.03)
who	60 (1.6)
lgbtq	50 (1.34)
aids	33 (0.88)
biden	30 (0.8)
cdc	30 (0.8)
billgatesbioterrorist	27 (0.72)
lgbt	23 (0.61)
trump	22 (0.59)
pandemic	22 (0.59)
vaccine	21 (0.56)
monkeypoxalypse	21 (0.56)
foxnews	20 (0.53)
pride	19 (0.51)
idiots	18 (0.48)

We also observed different types of entities in the hashtags relating to health administration organizations (eg, “who” and “cdc”), impactful individuals (eg, “biden” and “trump”), and news agencies (eg, “foxnews”). These hashtags indicate that further identification of users in this Twitter collection can help understand different stakeholders and participants in health and crisis information diffusion. Meanwhile, except for the query word “monkeypox,” all other hashtags were used <107 times. The variety of hashtags used and their low individual frequencies suggest that hashtags should not be the only source of identifying topics for this corpus. Alternative methods, such as topic modeling directly from the tweets, can be useful.

### Analytical Methods

#### Overview

In this section, we provide the details of the analytical framework for toxicity on social media in our analysis. We also illustrate the characterization of topical and network patterns in the toxic tweets on mpox. We first demonstrate the technical details of topic modeling. We then discuss social network analysis methods for analyzing toxic information diffusion.

#### Adaptation of an Analytical Framework of Hate Speech Analysis for Toxicity on Social Media

We leveraged the hate speech analysis by the Rabat Plan of Action [12,43] and adapted it to the context of toxicity on social media. Our adapted analytical framework includes the following 5 dimensions:

- Context—the social and public health landscape behind toxic online discourse, including what
  events
  co-occur during the discourse and how
  public health metrics
  change relative to the volume and content trends
- Extent—the severity to which the message can be considered
  abusive or harmful
  to the targeted group, which can be assessed with a score from 0 to 1
- Content—the semantic summary of toxic online discourse, which reveals
  attributes of the targeted group
  (eg, vulnerability, political representation, and social construct) and the discourse’s
  co-occurrence with other narratives
  that are dominant in toxic discourse (ie, major semantic clusters in the corpus)
- Speaker—the status of the social media user who posts toxic content, which can influence the
  dissemination quantity
  (indicated by network metrics) and
  quality
  (depending on the user’s credibility, influence, and capacity)
- Intent—the assumed high-level summary of
  objectives
  and
  intended audience
  for creating and spreading toxic content

These 5 dimensions are fundamental for our analysis and can benefit different stakeholders. Analyzing the context of toxic discourse can help policy makers identify key events that may be contributing to the proliferation of harmful messages, enabling them to address misinformation and foster a more supportive public health environment. Assessing the extent of toxic messages can help public health officials allocate resources and target interventions to counter the most severe cases of online abuse. Evaluating the content of toxic discourse reveals the attributes of targeted groups and dominant narratives, which can inform the development of tailored public health campaigns and interventions. Examining the speaker dimension provides insights into the dissemination of toxic content, allowing officials to monitor influential sources and mitigate their impact. Finally, understanding the intent behind toxic content can help public health policy makers craft strategies to engage with diverse audiences and counteract the harmful consequences of such discourse. By examining these 5 dimensions collectively, public health officials can gain a comprehensive understanding of the online toxic landscape during health crises, allowing them to devise timely and effective policy interventions. Table 2 further compares these 5 dimensions for analyzing toxic information diffusion with the original hate speech analysis framework by the Rabat Plan of Action [12].

**Table 2:** A comparison of analytical frameworks.

Dimension	Original definition	Definition for toxicity on social media in health crises	Analysis of adaptation
Context	Context denotes the social, cultural, and political landscape where the target of the hate speech is vulnerable.	It is defined as the social and public health landscape behind toxicity on social media, including what *events* co-occur during the discourse and how *public health metrics* change relative to the volume and content trends.	We limited the scope to social and public health and refocused the context from only the targeted group to the health crisis–related sociality.
Extent	Extent denotes the magnitude of the dissemination efforts or the extent of the hate speech act.	It is defined as the severity to which the message can be considered abusive or harmful to the targeted group, which can be assessed with a score from 0 to 1.	We measured the semantic intensity instead of the diffusion extent. We used Perspective API^a^ [9] to score toxicity. Diffusion is analyzed through the concept of “Speaker” in our definition.
Content	Content and form include the provocative degree or aggressiveness of the message, the form taken by the expression, directness, call to action degree, correlation with other dominant hate narratives, and legal status.	It is defined as the semantic summary of toxic online discourse, which reveals attributes of the targeted group (eg, vulnerability, political representation, and social construct) and the discourse’ co-occurrence with other narratives that are dominant in toxic discourse (ie, major semantic clusters in the corpus).	We kept 2 relevant subdimensions from the original definition.
Speaker	This dimension denotes the influence the speaker has on the audience to whom the SMS text message has been presented, including status, capacity, credibility, and influence on the targeted group.	It is defined as the status of the social media user who posts toxic content, which can influence the dissemination quantity (indicated by network metrics) and quality (depending on the user’s credibility, influence, and capacity).	We summarized the original definition into 2 aspects, namely, quantity and quality, which are about the speaker’s influence.
Intent	The intent of the speaker is estimated from past actions, reactions after promoting the hate message, probable objectives, and the intended audience.	It is defined as the assumed high-level summary of objectives and intended audience for creating and spreading toxic content.	We directly used the original definition in our context.
Likelihood of immediate actions	This dimension denotes the likelihood of the speech act generating a situation that represents a clear and immediate danger to the targeted social group, which is useful to evaluate as being sufficiently extreme to require a criminal investigation of censorship from state institutions.	—^b^	Our purpose was not to intervene in toxicity on social media as independent researchers but rather to identify and analyze them. Thus, the abovementioned 5 dimensions were sufficient.

^a^API: application programming interface.

^b^Not applicable.

### Modeling Topics in Toxic Online Discourse

Topic modeling is a method for detecting and analyzing latent semantic topics from large volumes of unstructured text data. It assumes that each text document (eg, a tweet) is a combination of multiple latent topics, where each topic is represented by a probability distribution of words while representing topics by grouping together words that have similar meanings based on their probability distributions [44,45]. Topic modeling identifies groups of words or vectors that appear together, and those groups are referred to as “topics.” They are not necessarily topics in the colloquial sense of a “subject” or “theme.” Identifying the content themes within and across topics requires manual inspection of the topics produced by the model. We refer to this step as “categorizing” and manually identified 5 themes (“categories”) that capture all 50 topics. Topic modeling and other semantic presentation methods have been used as big data analysis tools in a variety of fields of research, including social media studies, health informatics, and crisis informatics [46-48].

**Figure 3:**
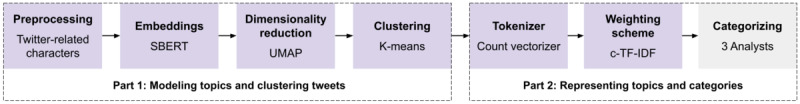
An extended sequence of steps with bidirectional encoder representations from transformers–based topic modeling technique (BERTopic). c-TF-IDF: class-based term frequency-inverse document frequency; SBERT: Sentence–bidirectional encoder representations from transformers; UMAP: uniform manifold approximation and projection.

With the development of deep learning techniques, such as transformers [49] and bidirectional encoder representations from transformers (BERT) [50], recent topic modeling methods take advantage of the embedding-based approach that better represents semantic relationships among words. These algorithms approach topic modeling as a clustering task and provide flexible language representation and text mining options [51,52]. As shown in Figure 3, our study follows this state-of-the-art development in topic modeling. We implemented a human-computer hybrid methodology sequence including both computational steps (in purple frames) and a human step (in a gray frame) for modeling topics in toxic online discourse during the mpox public health emergency. We followed the default steps and setting in BERT-based topic modelling technique (BERTopic) [52], which is a neural topic modeling method with a class-based term frequency-inverse document frequency (c-TF-IDF) procedure. We also extended the standard procedure by adding the preprocessing and the categorizing steps.

The first part of the BERTopic methodology sequence is modeling topics and clustering tweets. We started with preprocessing the toxic mpox tweets by removing Twitter-related characters, including “@” and “RT” marks for social media networks and links starting with “http” for external web information. We then used Sentence-BERT, a transformer-based pretrained natural language processing (NLP) model, to derive semantically meaningful sentence embeddings for each of the cleaned tweets [53]. In particular, we used the Sentence-BERT Python package and the pretrained model “all-MiniLM-L6-v2” [54], which enables clustering and semantic searching by mapping search tweets to a 384-dimensional vector space, making it effective for semantic similarity tasks. To better handle the high-dimensional tweet vectors for clustering, we implemented a dimensionality reduction technique (uniform manifold approximation and projection [UMAP]) [55]. UMAP helps cluster models handle dimensionality [56] while maintaining a dataset’s local and global structure. This feature of UMAP is important for constructing topic models, which depend on word vectors’ structural similarities.

We then used the scikit-learn implementation of the k-means clustering algorithm by Lloyd to group similar sentences’ embedding vectors into topics [57]. We used k-means clustering because it ensures that every vector is clustered into a topic and allows to select the number of clusters through experimentation. It first initiates centroids in the 384-dimensional vector space and randomly assigns a centroid to each of the 43,521 vectors. It then uses Euclidean distance [58] to update the centroid assignments recursively, which stops when the centroid assignments no longer update. We experimented with 3 different numbers of clusters: 30, 50, and 100. A total of 30 clusters produced clusters with a mix of topics and prevented us from disambiguating them. A total of 100 clusters were produced, resulting in sparse clusters that required manual combination of many topics. We settled on 50 clusters as a compromise. While the sentence embeddings remained the same, the clustering results could slightly vary with different random seeds initiated in the background. As there were only trivial differences among the clusters of sentences, we picked one of the results for our analysis. We shared the model on Hugging Face (Hugging Face, Inc) [59].

The second part of the sequence is representing topics and categories. We first tokenized topics using the count vectorizer in the *scikit-learn* Python package, which performs cluster-level (topic-level) bag-of-words representation that calculates and vectorizes the frequency of each word in each cluster [60]. In this study, we obtained word frequency vectors for each topic for representation purposes (ie, extracting keywords in each topic). This is fundamentally different from bag-of-words topic modeling, which uses corpus-level frequencies for creating topics. We then used c-TF-IDF to extract the difference of topical keywords, which helped distinguish among the clusters. After converting each cluster (topic) into a single document, we extracted the frequency of word *x* in class c [52]. In c-TF-IDF, we then had the importance score per word in each class:

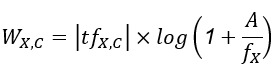



**(1)**

where *tf_X,C_* is the frequency of word in class *c, f_x_* is the frequency of word *x* across all classes, and *A* is the average number of words per class. In this way, we were able to represent topics with unique and frequent words as the keywords.

We then characterized the 50 topics based on the keywords and original tweets. After reviewing related literature, iterative refining categories, and labeling samples to study each category, we annotated each topic with the following 5 categories: disease, health policy and health care, homophobia, politics, and racism.

### Measuring User Influence in the Toxic Tweet Networks

Understanding how toxic information spreads on social media during public health crises is critical. Relationships, or user interactions, in social networks are often used to facilitate understanding of information diffusion in infodemiology [61]. We focused on 2 types of relationships on Twitter: mentions and retweets. A mention (ie, @username) is a tweet that quotes another user’s name in the text. The user who is mentioned will receive a notification from Twitter. A retweet is a reposting of a tweet that starts with “RT @username” [62]. We calculated 3 measures of influence in the network, including in-degree centrality, outdegree centrality, and betweenness centrality, with close-degree centrality as a representative metric. Degree centrality refers to the number of edges a vertex has to other vertices, and it defines 3 types of centrality [63]. In our study, we particularly focus on (1) in-degree centrality, which measures the number of incoming connections a node has, indicating its popularity or influence within the network; and (2) betweenness centrality, which assesses the extent to which a node acts as a bridge along the shortest path between other nodes. Given a network *G=(V,E)* with *V* vertices and E edges (defined as *deg(v)*), in-degree can be computed as [63] *deg_1(v)_ = N_V_* **(2)**

where N_V_ denotes the total number of all the incoming edges into vertex *v*. The betweenness of vertex *v* in a network is the fraction of all shortest paths between every pair of other vertices (*s, t*) that pass through vertex *v*. This is computed in three steps: (1) for each pair of vertices (*s, t*), shortest path between them is computed; (2) for each pair of vertices (*s, t*), the fraction of the shortest paths that pass through the vertex *v* is determined; and (3) the fraction over all pairs of vertices (*s, t*) is summed. Then, the betweenness of the vertex is calculated as [63]:

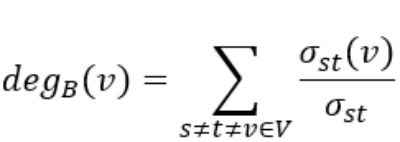



**(3)**

where *σ_st_* represents the total number of shortest paths from vertex s to vertex t, *σ_st_ (v)* is the number of those shortest paths that pass through vertex *v*. Further details of network analysis and metric calculation are provided in Multimedia Appendix 1.

Next, we applied a tool called NodeXL (Social Media Research Foundation) to generate the social network [64]. NodeXL is a visualization tool for social network analysis that is implemented as an add-on in Excel (Microsoft Corp) [64]. We further applied the Clauset, Newman, and Moore (CNM) algorithm [65] to investigate the social network in communicating toxicity on Twitter. The CNM algorithm infers the community structure from network topology that works by optimizing the modularity. It also provides insights into how vertices in social networks function and affect each other. One issue addressed by the CNM algorithm was to understand opinion leaders (eg, opinion leaders had high in-degree centrality) and distributors (eg, distributors had high betweenness centrality) in disseminating information.

As such, we generated social networks using mentions and retweets and investigated the following metrics in each network. The statistics for social networks *M* and *R* are summarized in Table 3 with the following attributes:

- Vertices—Twitter users in the social network
- Edges—relationships between 2 Twitter users (ie, retweet and mention)
- Duplicated edges—mention or retweet multiple times between 2 same Twitter users
- Self-loops—users mention or retweet their own tweets that form self-loops
- Connected components—a set of users in the network who are linked to each other by edges (ie, clusters in the social network)
- Geodesic distance—the length of the number of edges of the shortest path between 2 Twitter users (ie, 2 vertices in the network)

**Table 3:** Social network statistics for mentions and retweets networks.

Network metric	Mentions network	Retweets network
Network type	Directed	Directed
Vertices, n	17,437	59,749
Total edges, n	15,085	62,493
Duplicated edges, n	750	1663
Unique edges, n	14,335	60,830
Connected components, n	3628	3015
Self-loops, n	5	375
Maximum geodesic distance, n	26	21
Average geodesic distance, n	8.041	5.336

## Results

### Topic Modeling and Categorization

In this section, we report the topic modeling and categorization results, including the overall composition, temporal patterns, and representative tweets in each category. We summarize the results of the 50 topics into 5 *toxicity categories*, that is, the toxicity about 5 topical discourses, including disease (20,281/43,521, 46.6%), health policy and health care (8400/43,521, 19.3%), homophobia (10,402/43,521, 23.9%), politics (2611/43,521, 6%), and racism (1784/43,521, 4.1%).

**Figure 4:**
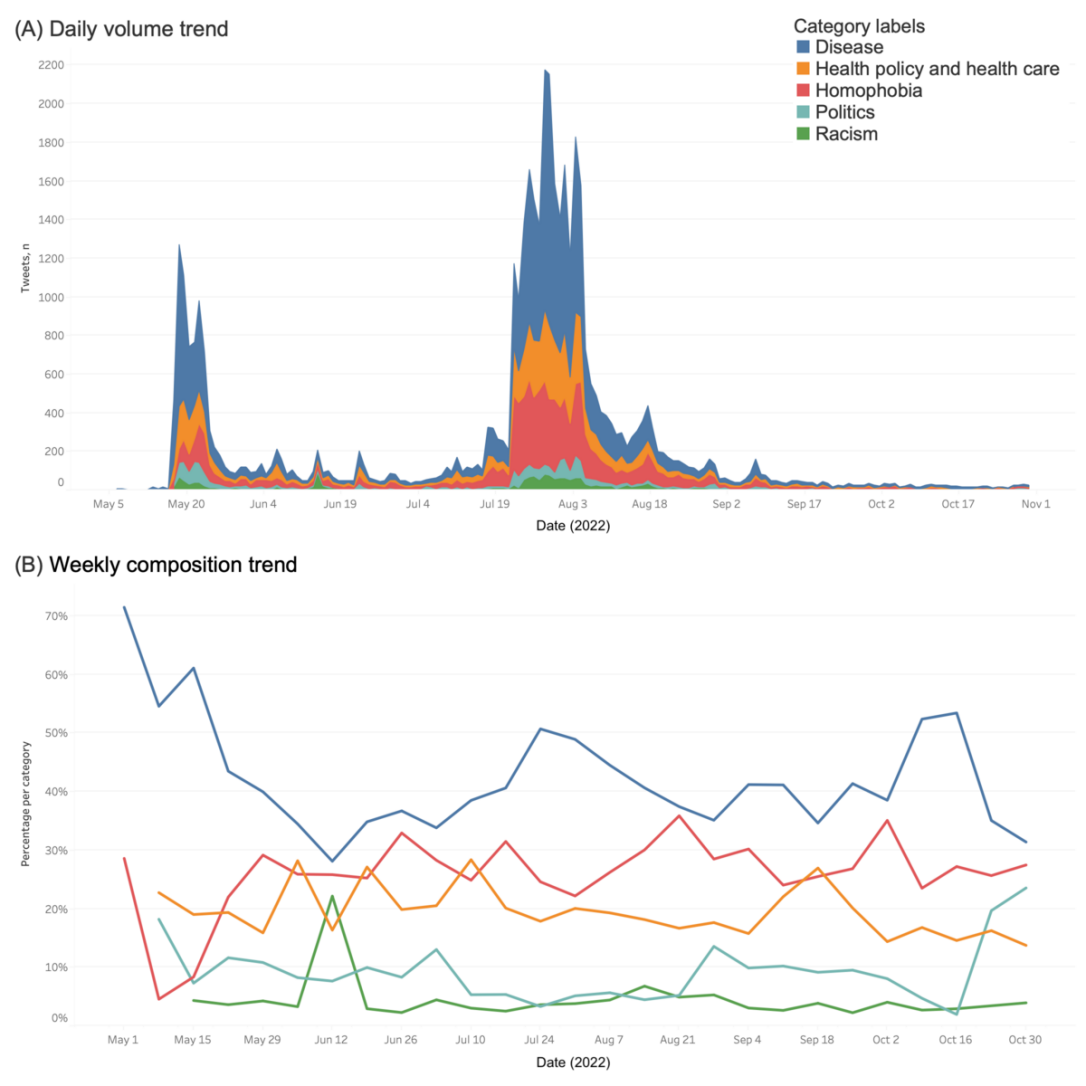
Overview of toxicity categories and change over time.

Figure 4 shows the daily volume trend and weekly composition trend as well as the composition overview of categories in toxic tweets during the mpox outbreak (from May 6 to October 31, 2022). The overall trend indicates that a wide range of topics exist in the semantic dispersion of tweets: except for the first week when only 2 categories of discourse occurred, we observed no discontinuity in any category, which shows strong topical diversity in toxic discourse.

Disease, health policy and health care, and homophobia were the dominant categories that had higher and comparatively stable compositions (often >10% of the overall discourse). At the same time, the politics and racism categories had lower daily volumes (often <100 tweets) and weekly compositions (often <10% of the discourse). These 2 categories also had more fluctuations in volume: there were continuous large discourses in the politics category between May 20 and June 4, 2022, and in August as well as a composition increase toward the end of October. There was also a large discourse in the racism category around May 20, 2022, and June 12, 2022, and between July 19 and August 23, 2022, with the cluster around June 12, 2022, comprising >20% of the discourse of that week.

**Figure 5:**
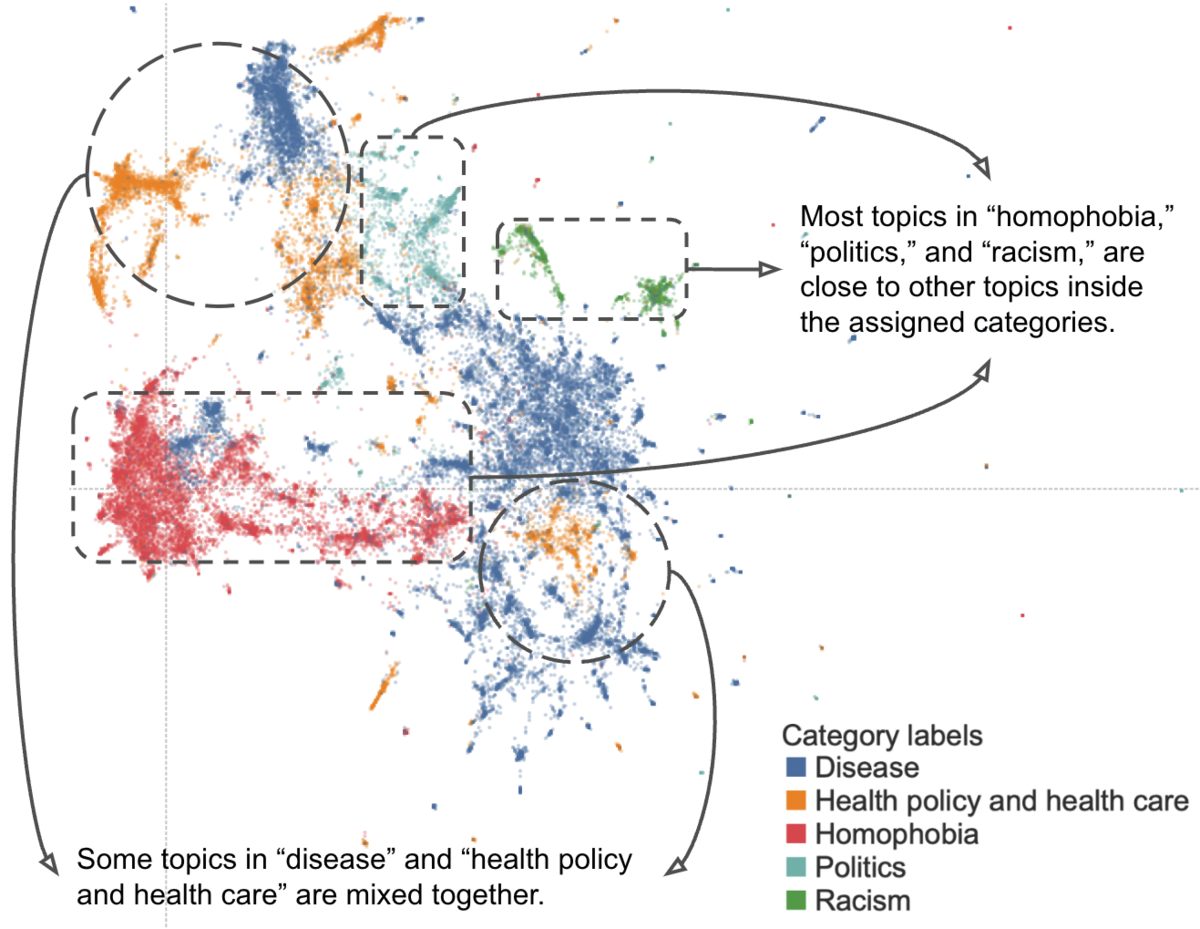
A 2D visualization of toxic tweets with categories.

Figure 5 demonstrates the interdocument and intercategory distances based on the UMAP mapping of tweets to the semantic spaces of topics in a 2D visualization [55], where the vertical and horizontal dashed lines are the axes of the 2 dimensions. The semantic space of toxic tweets is divided into several clusters, primarily based on the summative categories of Twitter topics. The overall dispersion of the topics shows that the related topics belonging to the same category are close to each other, which indicates the comprehensiveness of the topic modeling and the categorization process. The collocation of topics further indicates internal relevance among toxic tweets of different topical focuses.

The disease category and the health policy and health care category are mixed together in multiple positions due to their topical relevance (highlighted in dashed circular frames in Figure 5). Practically, negative emotions about the disease could either be amplified or mitigated depending on the effectiveness of health policies and services, which is the reason why the topics in these 2 categories are visually close. It is often difficult to talk about health care without talking about disease and illness because they are inherently related. Homophobia, politics, and racism categories are enclosed in connected areas, which demonstrate the different sociopolitical focuses in their individual discourse (highlighted in a dashed rectangular frame in Figure 5). To better demonstrate the categorization criteria and contents in each category, we also provide example tweets in Table 4 and more examples with notes in Tables S1-S5 in Multimedia Appendix 2.

**Table 4:** Example toxicity categories, topics, keywords, and tweets.

Category	Topics, n (%)	Keywords	Example tweet
Disease	6 (12)	scary_scared_shit_scaring	“If monkeypox was a person lol I swear that face kills me but no more mate, he’s scary as f*ck^a^ lol”
Health policy and health care	12 (24)	health_emergency_outbreak_cdc	“Hey CDC, F*ck You and your #monkeypox”
Homophobia	7 (14)	sex_anal_transmitted_spread	“Monkeypox is very serious, as serious as HIV for gay men having anal sex. The rest of us are Ok Follow Health Guidelines... avoid anal sex with gay men. Listen to the science! Nuff said? #Canada”
Politics	11 (22)	biden_ukraine_gates_f*ck	“You bet your ass they will.. School shooting, monkeypox. Magically the story has changed away from Biden and his sh*tty gas prices, baby formula shortages, massive inflation, etc”
Racism	18 (36)	n*gg*s_n*gg*_finna_yall	“Laughing at someone catching monkeypox. You n*gg*s are lame frfr”

^a^The vowels in inappropriate words are masked; emojis and some special characters are removed; user names are removed; and some capital and lower-case letters, spaces, and punctuations are adjusted.

### Information Diffusion Network

**Figure 6:**
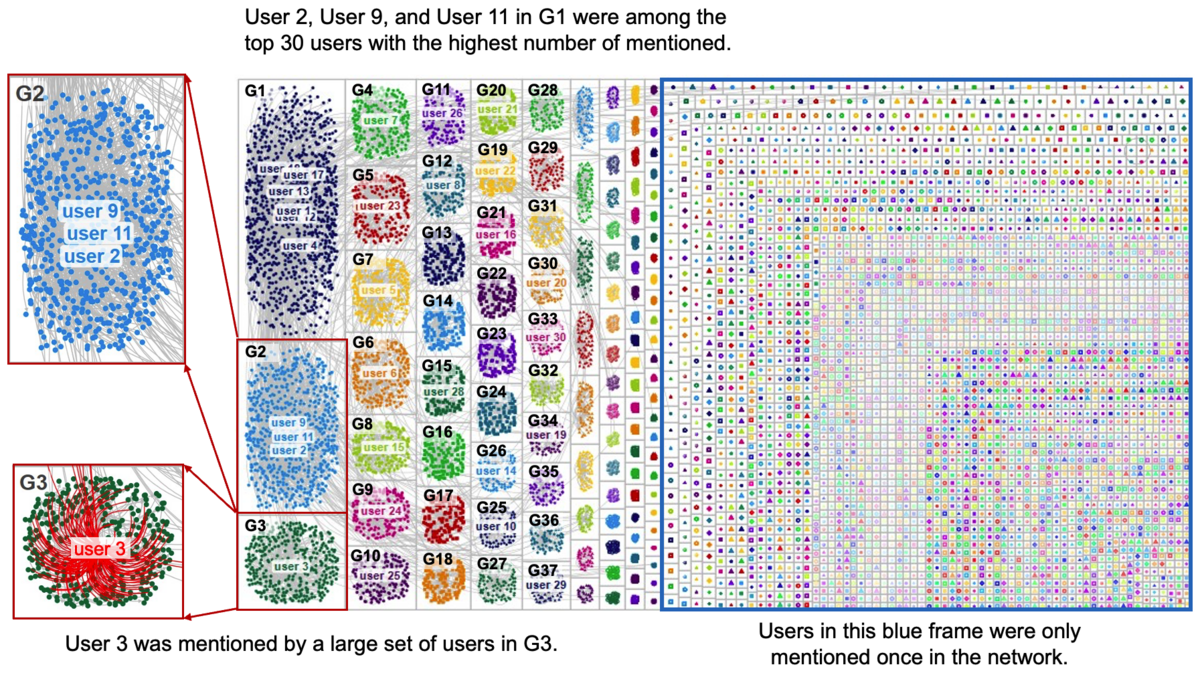
Network of Twitter users based on mentions. Each group number starts with a “G”.

**Figure 7:**
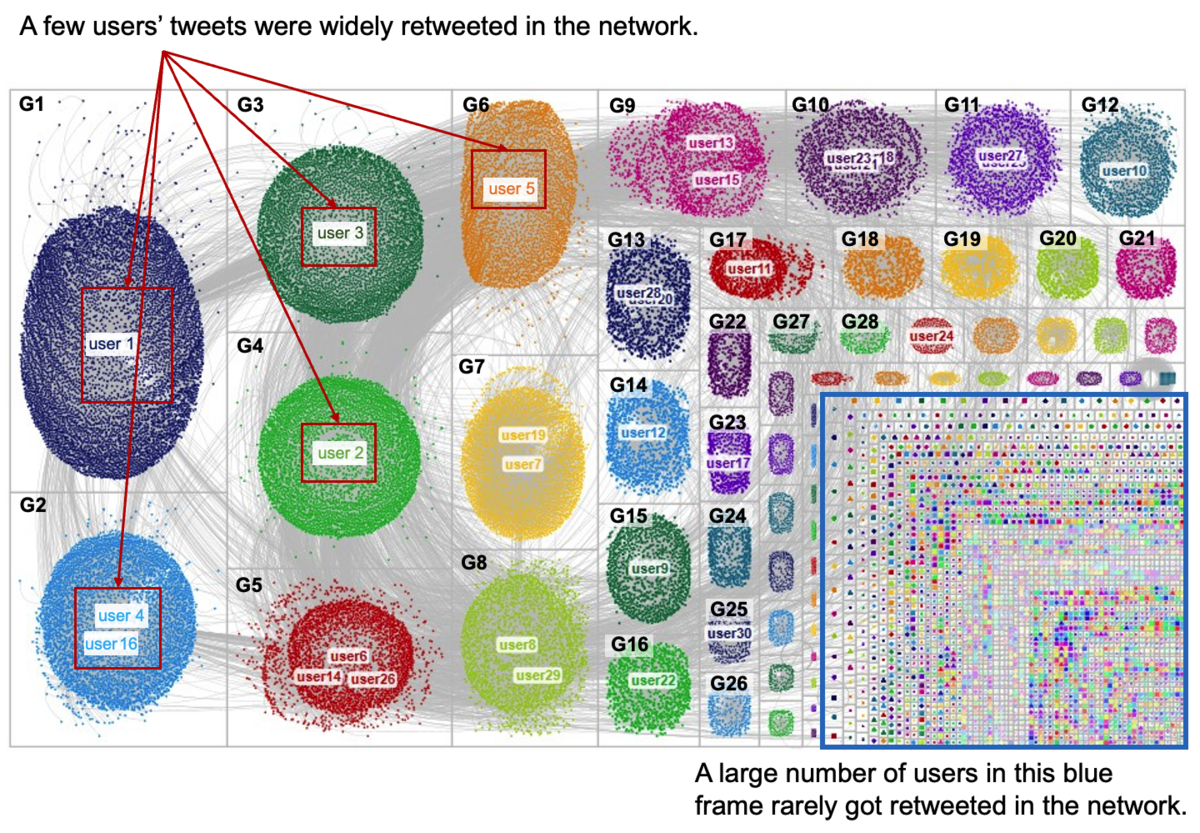
Network of Twitter users based on retweets. Each group number starts with a “G”.

As mentioned in the Analytical Methods section, we focused on 2 relationships on Twitter: mentions and retweets. The mentions network aimed to reveal which accounts were frequently mentioned and so were encouraged to respond, while the retweet network aimed to reveal which accounts diffused toxicity. The research objective was to locate users who perpetuated or spread toxicity in the network. The mentions network (Figure 6) included 17,437 vertices, 15,085 edges, and 3,628 connected components (ie, clusters in the network). The average geodesic distance between 2 vertices was 8.041. The retweets network (Figure 7) included 59,749 vertices, 62,493 edges, and 3015 connected components. The average geodesic distance was 5.336. Overall, the mentions network had fewer vertices, edges, and connected components but a greater geodesic distance than the retweets network. This observation implies fewer interactions between users in the mentions network, possibly because those mentioned users did not respond to such toxic comments.

Regarding the users, we observed that a few users dominate the network, as centered in the cluster and surrounded by a large set of users in Figures 6 and 7. These “centered” users were frequently mentioned or retweeted by other users in the community and thus had the highest in-degree centrality. For some clusters, 1 user dominated the entire cluster (eg, user 3 in cluster G3 in Figure 6). For other clusters, several users colocated in the same cluster (eg, user 2, user 9, and user 11 in cluster 2 in Figure 6), implying that their tweets shared similar outreaches and responses. We also observed that the users in the mentions network were more dispersed than those in the retweets network, as illustrated by a greater geodesic distance and a higher ratio of connected components divided by vertices. This reflects that a few users’ tweets are repeatedly retweeted in the community, but users’ mentions are arbitrary. However, many users were only mentioned or retweeted once according to both networks.

We further listed the top 30 users with the highest in-degree centrality with their account types in Tables 5 and 6. We used 2 attributes to describe a Twitter account. One attribute was the verification. A verified account may be an account of public interest, such as government agencies, politics, journalism, media, and influential public figures. For the verified accounts, we provided their usernames, while the usernames for nonverified accounts were masked. The other attribute was their account type. We manually interpreted each top user’s account description and classified it into one of the following categories, as listed below:

- Organization—news media (org_media) and government agencies (org_government)
- Individual users—politician (ind_politician), journalists (ind_journalist), high-impact users (ind_impact), and other users (ind_other)

We differentiated an individual account as a “high-impact user” or “other user” account based on its number of followers; an account with >50,000 followers is identified as a “high-impact user” account.

**Table 5:** Top 30 users based on in-degree centrality in the mentions network.

Username	In-degree centrality, n	Cluster	Verified status	Account type	Occurrence of each category, n
User 1 (POTUS)	229	G1	True	org_government	*D^a^* *(68), H^b^* *(47), O^c^* *(67), P^d^* *(46), and R^e^* *(2)*
User 2 (WHO)	201	G2	True	org_government	*D (57), H (39), O (46), *P* (34), and R (35)*
User 3 (RepMTG)	155	G3	True	ind_politician	*D (49), H (9), O (67), *P* (31), and R (1)*
User 4 (CDCgov)	153	G1	True	org_government	*D (44), H (50), O (49), *P* (14), and R (1)*
User 5 (TimRunsHisMouth)	115	G7	True	ind_impact	*D (36), H (12), O (66), *P* (8), and R (5)*
User 6 (FoxNews)	111	G6	True	org_media	*D (37), H (14), O (33), *P* (18), R and (13)*
User 7	80	G4	False	org_media	*D (26), H (12), O (31), *P* (10), and R (1)*
User 8 (nypost)	68	G12	True	org_media	*D (10), H (10), O (42), *P* (9), and R (4)*
User 9 (CNN)	67	G2	True	org_media	*D (19), H (5), O (34), *P* (9), and R (5)*
User 10	63	G25	False	ind_impact	*D (17), H (4), O (37), and *P* (6)*
User 11 (DrTedros)	61	G2	True	ind_politician	*D (22), H (12), O (16), *P* (7), and R (6)*
User 12 (JoeBiden)	59	G1	True	ind_politician	*D (24), H (12), O (9), *P* (12), and R (1)*
User 13 (CDCDirector)	57	G1	True	org_government	*D (16), H (21), O (14), *P* (7), and R (1)*
User 14 (SkyNews)	50	G26	True	org_media	*D (21), H (2), O (13), *P* (5), and R (12)*
User 15 (MrAndyNgo)	46	G8	True	ind_journalist	*D (16), H (4), O (26), *P* (2), and R (2)*
User 16 (JackPosobiec)	44	G21	True	ind_politician	*D (13), H (4), O (24), *P* (3), and R (1)*
User 17 (DrEricDing)	41	G1	True	ind_impact	*D (20), H (8), O (15), and *P* (3)*
User 18 (nytimes)	40	G1	True	org_media	*D (11), H (6), O (18), *P* (7), and R (1)*
User 19 (thehill)	40	G34	True	org_media	*D (13), H (5), O (20), *P* (4), and R (2)*
User 20	39	G30	False	org_media	*D (12), H (5), O (16), *P* (5), and R (1)*
User 21 (Reuters)	38	G20	True	org_media	*D (12), H (13), O (13), *P* (3), and R (12)*
User 22 (Timcast)	38	G19	True	ind_journalist	*D (18), H (4), O (13), *P* (1), and R (2)*
User 23 (newsmax)	37	G5	True	org_media	*D (18), H (4), O (11), *P* (2), and R (1)*
User 24 (washingtonpost)	36	G9	True	org_media	*D (7), H (3), O (24), *P* (1), and R (1)*
User 25 (Scott_Wiener)	35	G10	True	ind_politician	*D (4), H (5), O (22), *P* (3), and R (1)*
User 26 (ZubyMusic)	35	G11	True	ind_impact	*D (12), H (4), O (19), and *P* (3)*
User 27 (BetoORourke)	35	G5	True	ind_politician	*D (14), H (1), O (11), and *P* (11)*
User 28 (bethanyshondark)	34	G15	True	ind_impact	*D (15), H (5), and O (15)*
User 29 (unusual_whales)	33	G37	True	org_media	*D (10), H (7), and O (16)*
User 30 (MattWalshBlog)	33	G33	True	ind_impact	*D (5), H (5), O (25), and R (1)*

^a^D: disease.

^b^H: health policy and health care.

^c^O: homophobia.

^d^P: politics.

^e^R: racism.

**Table 6:** Top 30 users based on in-degree centrality in the retweets network.

Username	In-degree centrality, n	Cluster	Verified status	Account type	Occurrence of each category, n
User 1	7107	G1	False	ind_other	D^a^ (1)
User 2 (TimRunsHisMouth)	5038	G4	True	ind_impact	D (1), H^b^ (1), and O^c^ (1)
User 3	4325	G3	False	ind_impact	D (1)
User 4	4072	G2	False	ind_impact	D (1)
User 5	3222	G6	False	ind_impact	D (1)
User 6	2258	G5	False	ind_impact	H (1)
User 7	1947	G7	False	ind_impact	D (1)
User 8	1000	G8	False	ind_impact	H (1) and R^d^ (1)
User 9	897	G15	False	ind_impact	D (1) and O (1)
User 10 (bahjarodriguez)	804	G12	True	ind_impact	D (1)
User 11	599	G17	False	ind_impact	D (1)
User 12 (jennawadsworth)	571	G14	True	ind_politician	D (1)
User 13	535	G9	False	ind_impact	D (1)
User 14	391	G5	False	ind_impact	D (1)
User 15 (DrEricDing)	345	G9	True	ind_impact	H (1)
User 16 (AngryBlackLady)	336	G2	True	ind_impact	D (1)
User 17	332	G23	False	ind_other	H (1) and O (1)
User 18	291	G10	False	ind_impact	H (1)
User 19	285	G7	False	ind_impact	D (1)
User 20	284	G13	False	ind_other	H (1)
User 21	282	G10	False	ind_impact	H (2)
User 22	272	G16	False	ind_politician	D (1), H (1), and P^e^ (1)
User 23	260	G10	False	ind_other	H (1)
User 24	259	G29	False	ind_impact	D (1)
User 25 (johncardillo)	258	G11	True	ind_impact	D (1)
User 26	256	G5	False	ind_impact	D (2)
User 27	254	G11	False	ind_impact	*P* (1)
User 28	237	G13	False	ind_impact	R (1)
User 29	232	G8	False	ind_impact	D (1)
User 30	227	G25	False	ind_other	O (1)

^a^D: disease.

^b^H: health policy and health care.

^c^O: homophobia.

^d^R: racism.

^e^P: politics.

The sum of mentions in each category may be larger than the in-degree centrality in Table 5 because the in-degree centrality is computed based on the unique edges between 2 users.

We noted a couple of observations regarding the top users in the networks. For the mentions network, 90% (27/30) of the top-mentioned users had verified accounts, primarily news agencies, government portals, politicians, and independent high-impact users. In particular, the most frequently mentioned accounts were from government portals or politicians. By contrast, for the retweets network, most top users had nonverified accounts (24/30, 80%), and they were independent influencers (23/30, 77%) with large followings (ie, >50,000 followers). There is also a clear distinction regarding the organization accounts between mentions and retweets networks. More than half of the most frequently mentioned accounts (16/30, 55%) were from organization accounts, but none were among the top users in the retweets network.

Regarding the categories, these top users were frequently mentioned in tweets relative to disease, health policy and health care, and homophobia but comparatively less mentioned in the categories of politics and racism. On the basis of the retweets network, those tweets discussing disease and health policy and health care were most likely to obtain attention from the online community, while the other 3 categories did not. We observed that the categories of homophobia, politics, and racism were more likely to be shorter lived and more locally lived than the categories of disease and health policy and health care toxicity because these topics rarely appeared among the top retweeted users. Toxic tweets in the categories of homophobia and racism did not receive much attention, given that categories of “O” and “R” were rarely mentioned in Table 6.

## Discussion

### Principal Findings

Toxicity on social media is widespread during health crises, with many individuals spreading misinformation, fear, and hatred [8,9]. This can undermine public health communication efforts and lead to confusion and anxiety among the public. The discussion of toxic narratives during the 2022 mpox outbreak is an example of controversy in public communication during health crises. Building on prior work that leverages either topic modeling or network analysis techniques [66-68], our study further demonstrates the value of combining topical and network analyses to understand emerging social issues and crises. By examining the topical dynamics, we were able to uncover the prevalent themes in the toxic discourse during the 2022 mpox outbreak and observe their temporal shifts. Network dynamics revealed the key users and their roles in propagating toxicity, suggesting that addressing these high-impact users and their narratives could be crucial for effective crisis communication and policy decision-making. Our findings highlight the importance of monitoring and addressing toxicity on social media to foster a more inclusive and constructive public dialogue during health emergencies.

We also adapted the Rabat Plan of Action analytical framework for hate speech analysis to study toxicity on social media during the mpox outbreak. This framework takes into account the context, extent, content, speaker, and intent. By examining the context of the discussions, including the events that led to the discourse, the extent of toxic comments and hate speech, the content and themes, the speakers involved, and the intent behind the messages, the adapted analytical framework provided a comprehensive understanding of the toxicity landscape in the mpox scenario.

### Toxicity Aboutness Reveals an Infodemic: Negative Feelings, Political Unrest, and Weaponized Stigma

Topical dynamics summarize temporal content popularity and provide an extended context of social issues in the mpox health crisis. In this sense, the understanding of context and content of toxicity represents the extremes of public opinion, respectively, from event and topical trend perspectives, which are mutually beneficial in profiling the problems in health communications during the 2022 mpox public health emergency. By examining groups of related topics, we can grasp an overarching view of the main subjects being discussed. In other words, we can identify the primary categories that highlight the most commonly mentioned topics. By analyzing temporal topical swifts, we further understand when the topical discourses, especially their peaks, occur in each of the categories. Such a summary of contents can reveal what categories of topics are discussed together, which quantitatively demonstrates public opinions around context and facilitates a multifaceted understanding of toxic contents in this health crisis. In particular, there are 3 outstanding problems associated with toxicity on social media: negative feelings, political unrest, and weaponized stigma.

Negative feelings during the 2022 mpox public health emergency often came out of emotions toward the disease or how the disease was dealt with. There were negative feelings, such as fear and anger, due to the actual or imagined physical symptoms caused by mpox or negativity because of mental anxiety. Specific to the 2022 mpox outbreak, there were also negative feelings carried on from the COVID-19 pandemic because some initial public health guidance were similar (eg, vaccination, wearing masks, and self-quarantine). Thus, similar to the COVID-19 pandemic, people could also be unhappy about how the health emergency was dealt with by the health authorities and health care providers. These negative feelings are not produced in isolation: Twitter users read and watch news from different media platforms and share their ideas on the platform. When some personal beliefs, which might not be scientifically mature, are put together with the practical inconvenience, the public uses toxicity to express their negativity toward the disease and the health services they receive.

Political unrest during the 2022 mpox outbreak was fueled by divisive reactions from politicians and the public, leading to the spread of conspiracy theories and misinformation. Disagreements over health policies, allocation of resources, and the overall handling of the outbreak often manifested in toxic discourse, further polarizing society. For example, some Twitter users propagated unfounded claims that the mpox outbreak was a result of a laboratory leak or a government conspiracy, which led to increased distrust in the authorities and health care providers. This toxic environment can be understood through the lens of psychological factors, such as fear, uncertainty, and a tendency toward confirmation bias, where individuals are more likely to believe and spread information that aligns with their pre-existing beliefs and fears. Societal factors, such as political polarization and a general erosion of trust in public institutions, also played a critical role in amplifying toxic behavior on the internet, as people sought out and shared content that validated their anxieties and skepticism.

Weaponized stigma became another significant issue during the outbreak, as incidents of attacks toward minority groups based on (perceived) sexuality, gender identity, and race were reported. This stigmatization was often rooted in misinformation and fear, with people associating certain groups with the spread of the disease or accusing them of not following public health guidelines. Toxicity on social media facilitated the perpetuation of these stigmatizing narratives, further marginalizing these communities and exacerbating existing social divisions. Psychological factors such as xenophobia, scapegoating, and the need to find a tangible source of blame during a crisis contributed to the spread of these harmful narratives. Societal factors, including systemic discrimination and historical prejudices, were also at play, as these pre-existing biases were amplified in the digital space, leading to more virulent expressions of hate and intolerance. Understanding these dynamics is crucial for developing strategies to mitigate toxicity on social media and support affected communities.

### Toxicity Diffusion Suggests Improvements in Health Communication: Influential Users Should Respond to and Counter Toxicity

Network dynamics reveal frequent speakers and their intentions, highlighting key priorities for public health communication and health policy. On the basis of the analytical framework, we used the social network theory to calculate the in-degree and betweenness centrality. Through this approach, we were able to identify influential speakers and mentioned users as well as gain insights into what they said and the likelihood of responses in online communities. Our analysis reveals several key observations regarding speakers and their intentions that are worth discussing.

In our analysis of speakers, we discovered that a few nonverified yet influential users dominated the retweets network. Their tweets garnered broad attention from the online community and resonated with many others who shared the same opinion regarding disease- and health-related negativity. By contrast, users who were mentioned in these toxic messages appeared to be scattered and chosen randomly, and they seldom responded to the negativity. Our findings suggest that toxic information, regardless of its intent, typically does not elicit responses from those who are mentioned.

For those most frequently mentioned users, our analysis revealed that verified government channels, news agencies, and politicians dominated the top-mentioned list. This finding highlights the importance of government and health agents being aware of toxic information and taking appropriate action. For example, some users expressed concerns on Twitter about the transmission routes of a disease, albeit in a toxic manner. To help limit such toxicity, we suggest that public health organizations such as the WHO and Centers for Disease Control and Prevention should inform the public about the transmission routes of the disease and the severity of the health crisis. This underscores the need for timely and effective communication from official sources in response to public concerns.

Upon analyzing intents in the retweets network, we observed that attributions of diseases to homophobia and racism were not frequently mentioned by the top speakers. While 1 interpretation could be that most online users view such attributions as malicious during public health crises, another perspective is that these top speakers, who are already prominent and attract attention regardless of their content, might not engage in such rhetoric. If these influential speakers had used this type of rhetoric, it is possible that it would have still received significant engagement. In contrast, tweets related to disease- and health care policy–related negativity were more generalized among a broader set of users and were retweeted for a more extended period. Moreover, our findings highlight the importance of focusing on accurate and relevant information during public health crises, as misinformation or toxic narratives can be quickly dismissed by online users. We suggest that public health agencies prioritize accurate information dissemination, which can help combat the spread of harmful narratives and promote healthy dialogue and public understanding of health-related issues.

After analyzing intents in the mentions network, we found that verified users were primarily mentioned in topics related to disease-related negativity, health policy–related negativity, and homophobia. Our findings reveal widespread dissatisfaction regarding health policies for mpox and concerns about the severity of the disease outbreak. This highlights the urgent need for government or health channels to release transparent and reliable information to address public concerns. Our findings also imply that some users might misunderstand the disease transmission or use mpox to stigmatize homosexuality. This indicates a need for relevant agencies to take immediate action to interrupt the dissemination of toxic information. By doing so, we can mitigate the influence of toxicity and reduce the harm to groups considered historically marginalized, such as the lesbian, gay, bisexual, transgender, queer, and questioning (LGBTQ+) community.

### Comparison to Prior Work

Previous research has demonstrated that NLP techniques, such as topic modeling [66] and text classification [69], could be useful for uncovering toxicity on social media and hate speech during health crises. In the broader context of health communication, our study highlights several significant implications. One key implication involves the use of NLP and computational techniques to analyze social media narratives. Our research further shows that combining topic modeling and network analysis can provide a nuanced understanding of online toxic narratives and their dissemination.

Next, our topic modeling results suggest common underlying causes of toxicity and hate speech on social media, including emotional, political, and stigmatizing responses. Toxic narratives by negative feelings have been widely reported in previous studies [70,71], often linked to policies such as lockdowns and mask mandates during the COVID-19 pandemic [72]. In addition, political unrest and misinformation exacerbate these emotions, with conspiracy theories and distrust in authorities intensifying toxic narratives. This pattern, observed during the mpox outbreak, was also prevalent in past pandemics, such as the COVID-19 pandemic [67]. Moreover, the stigmatization of certain groups, such as the gay community during the mpox outbreak, mirrors the scapegoating and xenophobia seen in previous crises. For instance, previous studies reported widespread anti-Asian sentiment on social media during the COVID-19 pandemic [73,74]. Understanding these causes in terms of misinformation, distrust of government or health agencies, and societal biases on certain groups is crucial for developing strategies to mitigate the harmful impact of toxicity on social media during health crises.

Our network analysis highlights the crucial need to engage with influential users and address key narratives to mitigate the spread of toxicity on social media during health crises. Consistent with previous research [32], our findings suggest that official sources, such as government agencies and health care organizations, must prioritize timely, transparent, and accessible communication across major social media platforms, as these entities are frequent targets of toxic discourse. While earlier studies have found that right-wing sources are often linked to higher levels of toxicity and scientific sources to lower levels [67], our research indicates that general users, rather than verified accounts, are more likely to lead and propagate toxic narratives. This underscores the importance of not only monitoring influential users but also addressing the broader network of general users who contribute to the dissemination of harmful content.

### Limitations and Outlook

One limitation could result from the use of Perspective API. For example, Perspective API might incorrectly identify the toxicity if a tweet’s toxic words or patterns do not appear similar to its training samples. In particular, social media language is informal, and Perspective API might not be able to identify internet slang or abbreviations correctly. In addition, the threshold selection may have impacted the toxicity results. A smaller threshold increases the likelihood of identifying a toxic tweet. However, it simultaneously increases the number of false positives (ie, a nontoxic tweet is identified as toxic) [10].

Another limitation relates to the types of toxicity used. Toxicity on social media may include both emphasis of emotions (eg, fear and anxiety) toward the crisis and attacks of associated minority groups (eg, Asian Americans during the COVID-19 pandemic and LGBTQ+ people during the 2020 mpox outbreak). There is no clear boundary between the users who spread these 2 types of toxicity or between the language they use. For example, while a significant portion of the toxicity targets LGBTQ+ or African and African American communities, it is difficult to determine if some tweets are using toxic language to highlight inequity or discrimination and condemn identity-based attacks. Future work could include an analysis of nontoxic discourse to serve as a comparison to facilitate the overall understanding of toxic tweets about mpox. By examining the overlap between the discourse in toxic versus nontoxic discourse, especially the influential users behind it, we can better understand the extent to which these users are involved in combating misinformation and toxic speech. This comparative analysis would provide a more comprehensive depiction of user discourse on social media, with a focus on showcasing the difference between emphasis and toxicity.

In addition, algorithmic bias is an important consideration in our study, particularly concerning the algorithms used for data analysis, such as BERT and UMAP. BERT, a transformer-based model, is pretrained on large text corpora, which may contain inherent biases reflecting societal stereotypes and prejudices. These biases can influence the model’s understanding and representation of language, potentially skewing the identification and clustering of topics. Similarly, UMAP, a dimensionality reduction technique, might introduce biases through its assumptions about data structure and the preservation of local and global relationships within the dataset. These algorithmic biases can impact the study’s findings by potentially misrepresenting the semantic relationships and topic distributions within the toxic discourse, leading to conclusions that may not fully or accurately reflect the underlying data. Acknowledging these biases is crucial, and future work should focus on using bias mitigation strategies, such as algorithmic auditing and using debiased training datasets, to enhance the fairness and accuracy of the analysis.

At the same time, toxicity can look different in different parts of the world, among different cultural groups, and in different languages. While our analysis focused on English-speaking countries, it is important to recognize that many affected populations are not represented in the tweets we collected. Our approach may inherently overlook the perspectives and nuances present in non-English tweets, which may result in a cultural bias. We chose English content primarily due to the availability of robust language processing tools and resources for English, which facilitates more reliable analysis. We recognize that this focus might limit the generalizability of our findings to non–English-speaking audiences, and we have listed this as a limitation in the Discussion. The discourse around public health crises, such as the mpox outbreak, is multifaceted and culturally dependent. Non-English content may reveal different concerns, misinformation patterns, and public reactions that our study does not capture. As such, we could investigate toxicity with more granularity in the future and characterize it with regard to attack versus emphasis as well as demographic factors, including language, country, and culture.

### Conclusions

Toxic online discourse can have detrimental impacts on public health crises. In this study, we collected tweet data during the 2022 mpox outbreak and analyzed toxicity from multiple dimensions, including context, extent, content, speaker, and intent. To better understand toxic dynamics on Twitter, we used BERTopic and social network community clustering techniques.

The temporal discourse analysis revealed that toxic tweets during the outbreak covered a diverse range of topical categories. The predominant topics were toxicity on disease, health policy and health care, and homophobia. While toxicity related to politics and racism had lower daily volumes, they reached respective peaks when triggering events happened. On the other hand, verified government channels, news agencies, and politicians were among the top-mentioned users in the social network and were primarily associated with the categories of disease, health policy and health care, and homophobia. Meanwhile, a few nonverified but influential users posted tweets that received high volumes of retweets, and tweets related to homophobia, politics, and racism were more likely to be shorter lived and have a local impact.

As such, to mitigate the toxicity on social media of mpox or similar infodemics, public health authorities should leverage advanced NLP tools, such as sentiment analysis and toxicity detection algorithms, to identify and address harmful content in real time. In addition, digital literacy campaigns can educate the public about the dangers of misinformation and the importance of respectful online discourse. Establishing rapid response teams comprising public health experts, communication specialists, and community leaders can help counteract false narratives and provide accurate information swiftly. Finally, fostering partnerships with influential social media figures and organizations can amplify positive messages and mitigate the spread of toxic content.

To summarize, the topical dynamics revealed that Twitter users were expressing negativity and making controversial remarks about the mpox public health emergency, indicating a worsening of political unrest and the increased weaponization of stigma during the corresponding infodemic. The network dynamics highlight the need for government and health agencies to release transparent and reliable information to address public concerns. Overall, our study demonstrates a workflow that combines topical and network analyses to understand emerging social issues and crises. Our findings emphasize the importance of proactive measures needed from government and health agencies to combat harmful narratives and promote accurate information during public health crises.

## Appendix

Multimedia Appendix 1 Method notes on network analysis.

Multimedia Appendix 2 Notes on topic modeling.

## Abbreviations

API: application programming interface
BERT: bidirectional encoder representations from transformers
BERTopic: bidirectional encoder representations from transformers–based topic modeling technique
CNM: Clauset, Newman, and Moore
c-TF-IDF: class-based term frequency-inverse document frequency
LGBTQ+: lesbian, gay, bisexual, transgender, queer, and questioning
NLP: natural language processing
UMAP: Uniform Manifold Approximation and Projection
WHO: World Health Organization

## Data Availability

The datasets generated during and analyzed during this study are not publicly available due to Twitter’s developer agreement and policy but are available from the corresponding author on reasonable request. Raw data, consisting of tweets and user information, cannot be shared directly due to privacy and data protection regulations. Note that the Twitter Academic application programming interface service was discontinued as of mid-2023, while access to Twitter data are still available at a cost [37].

## References

[1] Posner L, Turilli I Monkeypox timeline: a frequently updated tracker of emerging developments from the beginning of the 2022 monkeypox outbreak. Think Global Health.

[2] Mpox (monkeypox). World Health Organization (WHO).

[3] WHO recommends new name for monkeypox disease. World Health Organization (WHO).

[4] Factsheet for health professionals on mpox (monkeypox). European Centre for Disease Prevention and Control.

[5] FDA Mpox response. U.S. Department of Food and Agriculture.

[6] HHS response to the Mpox outbreak. Assistant Secretary for Public Affairs (ASPA), U.S. Department of Health and Human Services.

[7] Edinger A, Valdez D, Walsh-Buhi E, Trueblood JS, Lorenzo-Luaces L, Rutter LA, Bollen J (2023). Misinformation and public health messaging in the early stages of the Mpox outbreak: mapping the Twitter narrative with deep learning. J Med Internet Res.

[8] Kolhatkar V, Wu H, Cavasso L, Francis E, Shukla K, Taboada M (2020). The SFU opinion and comments corpus: a corpus for the analysis of online news comments. Corpus Pragmat.

[9] Using machine learning to reduce toxicity online. Perspective.

[10] Rieder B, Skop Y (2021). The fabrics of machine moderation: studying the technical, normative, and organizational structure of Perspective API. Big Data Soc.

[11] Turner A (2023). How many users does X (formerly Twitter) have?. Bankmycell.

[12] The Rabat plan of action. United Nations Human Rights.

[13] Sheth A, Shalin VL, Kursuncu U (2022). Defining and detecting toxicity on social media: context and knowledge are key. Neurocomputing.

[14] TDL brief: online toxicity. The Decision Lab.

[15] Suler J (2004). The online disinhibition effect. Cyberpsychol Behav.

[16] Shen C, Sun Q, Kim T, Wolff G, Ratan R, Williams D (2020). Viral vitriol: predictors and contagion of online toxicity in world of tanks. Comput Human Behav.

[17] Kim JW, Guess A, Nyhan B, Reifler J (2021). The distorting prism of social media: how self-selection and exposure to incivility fuel online comment toxicity. J Commun.

[18] Parent MC, Gobble TD, Rochlen A (2019). Social media behavior, toxic masculinity, and depression. Psychol Men Masc.

[19] Wijesiriwardene T, Inan H, Kursuncu U, Gaur M, Shalin VL, Thirunarayan K, Sheth A, Arpinar IB (2020). ALONE: a dataset for toxic behavior among adolescents on Twitter. Proceedings of the 12th International Conference on Social Informatics.

[20] Suarez Estrada M, Juarez Y, Piña-García CA (2022). Toxic social media: affective polarization after feminist protests. Soc Media Soc.

[21] Guberman J, Schmitz C, Hemphill L (2016). Quantifying toxicity and verbal violence on Twitter. Proceedings of the 19th ACM Conference on Computer Supported Cooperative Work and Social Computing Companion.

[22] Almerekhi H, Kwak H, Jansen BJ, Salminen J (2019). Detecting toxicity triggers in online discussions. Proceedings of the 30th ACM Conference on Hypertext and Social Media.

[23] Lwin MO, Lu J, Sheldenkar A, Schulz PJ, Shin W, Gupta R, Yang Y (2020). Global sentiments surrounding the COVID-19 pandemic on Twitter: analysis of Twitter trends. JMIR Public Health Surveill.

[24] Moderation. OpenAI.

[25] Record I, Miller B (2022). People, posts, and platforms: reducing the spread of online toxicity by contextualizing content and setting norms. Asian J Philos.

[26] Salminen J, Sengün S, Corporan J, Jung SG, Jansen BJ (2020). Topic-driven toxicity: exploring the relationship between online toxicity and news topics. PLoS One.

[27] Denecke K, Atique S, Syed-Abdul S, Gabarron E, Lau AY (2016). Social media and health crisis communication during epidemics. Participatory Health Through Social Media.

[28] Sellnow TL, Sellnow DD, Helsel EM, Martin JM, Parker JS (2018). Risk and crisis communication narratives in response to rapidly emerging diseases. J Risk Res.

[29] Gesser-Edelsburg A, Shir-Raz Y, Walter N, Mordini E, Dimitriou D, James JJ, Green MS (2015). The public sphere in emerging infectious disease communication: recipient or active and vocal partner?. Disaster Med Public Health Prep.

[30] van Velsen L, van Gemert-Pijnen JE, Beaujean DJ, Wentzel J, van Steenbergen JE (2012). Should health organizations use web 2.0 media in times of an infectious disease crisis? An in-depth qualitative study of citizens' information behavior during an EHEC outbreak. J Med Internet Res.

[31] Majó-Vázquez S, Nielsen RK, Verdú J, Rao N, De Domenico M, Papaspiliopoulos O Volume and patterns of toxicity in social media conversations during the COVID-19 pandemic. Reuters Institute for the Study of Journalism.

[32] Pascual-Ferrá P, Alperstein N, Barnett DJ, Rimal RN (2021). Toxicity and verbal aggression on social media: polarized discourse on wearing face masks during the COVID-19 pandemic. Big Data Soc.

[33] Kim DK, Kreps GL (2020). An analysis of government communication in the United States during the COVID-19 pandemic: recommendations for effective government health risk communication. World Med Health Policy.

[34] Zarocostas J (2020). How to fight an infodemic. Lancet.

[35] Eysenbach G (2020). How to fight an infodemic: the four pillars of infodemic management. J Med Internet Res.

[36] Covello VT (2003). Best practices in public health risk and crisis communication. J Health Commun.

[37] Twitter academic API. Twitter.

[38] Bryant M Patient treated for monkeypox in isolation at London hospital. The Guardian.

[39] Mathieu E, Spooner F, Dattani S, Ritchie H, Roser M (2022). Mpox (monkeypox). Our World in Data.

[40] Hermann P (2022). Police: assailants used slur and referenced monkeypox in Shaw attack. The Washington Post.

[41] Kates J, Artiga S, Dawson L National data show continuing disparities in Monkeypox (MPX) cases and vaccinations among black and Hispanic people. Kaiser Family Foundation (KFF).

[42] As Monkeypox eases in the West, "nothing has changed" for Africa, where doctors remain helpless. CBS News.

[43] Toolkit for analysing a case of hate speech. European Court of Human Rights, The European Union.

[44] Blei DM, Lafferty JD (2007). A correlated topic model of science. Ann Appl Stat.

[45] Steyvers M, Griffiths T, Landauer TK, McNamara DS, Dennis S, Kintsch W (2007). Probabilistic topic models. Handbook of Latent Semantic Analysis.

[46] Manna S, Phongpanangam O (2018). Exploring topic models on short texts: a case study with crisis data. Proceedings of the 2018 Second IEEE International Conference on Robotic Computing.

[47] Yu H, Fan L, Gilliland AJ (2022). Disparities and resilience: analyzing online health information provision, behaviors and needs of LBGTQ + elders during COVID-19. BMC Public Health.

[48] Li L, Ma Z, Fan L, Lee S, Yu H, Hemphill L (2023). ChatGPT in education: a discourse analysis of worries and concerns on social media. Educ Inf Technol.

[49] Vaswani A, Shazeer N, Parmar N, Uszkoreit J, Jones L, Gomez AN, Kaiser L, Polosukhin I (2023). Attention is all you need. arXiv. Preprint posted online August 2, 2023.

[50] Devlin J, Chang MW, Lee K, Toutanova K (2019). BERT: pre-training of deep bidirectional transformers for language understanding. arXiv. Preprint posted online May 24, 2019.

[51] Sia S, Dalmia A, Mielke SJ (2020). Tired of topic models? Clusters of pretrained word embeddings make for fast and good topics too!. Proceedings of the 2020 Conference on Empirical Methods in Natural Language Processing.

[52] Grootendorst M (2022). BERTopic: neural topic modeling with a class-based TF-IDF procedure. arXiv. Preprint posted online March 11, 2022.

[53] Reimers N, Gurevych I (2019). Sentence-BERT: sentence embeddings using Siamese BERT-networks. arXiv. Preprint posted online August 27, 2019.

[54] Cuenca NR all-MiniLM-L6-v2. Hugging Face.

[55] McInnes L, Healy J, Saul N, Großberger L (2018). UMAP: uniform manifold approximation and projection. J Open Source Softw.

[56] Indyk P, Motwani R (1998). Approximate nearest neighbors: towards removing the curse of dimensionality. Proceedings of the 13th annual ACM symposium on Theory of computing.

[57] sklearn.cluster.KMeans. Scikit-learn.

[58] Danielsson PE (1980). Euclidean distance mapping. Comput Graph Image Process.

[59] Fan L lizhouf/mpox_50_topics_modeling. Hugging Face.

[60] Zhang Y, Jin R, Zhou ZH (2010). Understanding bag-of-words model: a statistical framework. Int J Mach Learn Cybern.

[61] Chu AM, Chong AC, Lai NH, Tiwari A, So MK (2023). Enhancing the predictive power of Google trends data through network analysis: infodemiology study of COVID-19. JMIR Public Health Surveill.

[62] About different types of posts. X (Formerly Twitter).

[63] Knoke D, Yang S (2008). Social Network Analysis.

[64] Smith M, Milic-Frayling N, Shneiderman B, Mendes RE, Leskovec J, Dunne C (2019). NodeXL: a free and open network overview, discovery and exploration add-in for Excel 2007/2010. ScienceOpen.

[65] Clauset A, Newman ME, Moore C (2004). Finding community structure in very large networks. Phys Rev E Stat Nonlin Soft Matter Phys.

[66] Obadimu A, Khaund T, Mead E, Marcoux T, Agarwal N (2021). Developing a socio-computational approach to examine toxicity propagation and regulation in COVID-19 discourse on YouTube. Inf Process Manag.

[67] Chipidza W (2021). The effect of toxicity on COVID-19 news network formation in political subcommunities on Reddit: an affiliation network approach. Int J Inf Manage.

[68] Alshalan R, Al-Khalifa H, Alsaeed D, Al-Baity H, Alshalan S (2020). Detection of hate speech in COVID-19-related tweets in the Arab region: deep learning and topic modeling approach. J Med Internet Res.

[69] Wani MA, ELAffendi M, Shakil KA, Abuhaimed IM, Nayyar A, Hussain A, El-Latif AA (2024). Toxic fake news detection and classification for combating COVID-19 misinformation. IEEE Trans Comput Soc Syst.

[70] Khaskheli A, Jiang Y, Raza SA, Qamar Yousufi S (2022). Social isolation and toxic behavior of students in e-learning: evidence during the time of the COVID-19 pandemic. Interact Learn Environ.

[71] Awal MR, Cao R, Mitrovic S, Lee RK (2020). On analyzing antisocial behaviors amid COVID-19 pandemic. arXiv. Preprint posted online July 21, 2020.

[72] DiCicco K, Noor N, Yousefi N, Maleki M, Spann B, Agarwal N (2023). Toxicity and networks of COVID-19 discourse communities: a tale of two social media platforms. Proceedings of the 3rd Workshop on Reducing Online Misinformation through Credible Information Retrieval, held as part of ECIR 2023: the 45th European Conference on Information Retrieval.

[73] Gover AR, Harper SB, Langton L (2020). Anti-Asian hate crime during the COVID-19 pandemic: exploring the reproduction of inequality. Am J Crim Justice.

[74] Fan L, Yu H, Yin Z (2020). Stigmatization in social media: documenting and analyzing hate speech for COVID-19 on Twitter. Proc Assoc Inf Sci Technol.

