# Supplementary material for: Toxicity on Social Media During the 2022 Mpox Public Health Emergency: Quantitative Study of Topical and Network Dynamics
Source: J Med Internet Res. 2024 Dec 12;26:e52997. doi: 10.2196/52997 (PMC11671789; doi:10.2196/52997)
Supplement: Multimedia Appendix 1 [file jmir_v26i1e52997_app1.docx]

## Method Notes on Network Analysis

We leveraged social network theory to discover toxic communication patterns during the Mpox crisis. **Figure S1** illustrates the relationships among Twitter users and the degree centrality theory: a. Two ways of communication on Twitter. b. An illustration of a Twitter network.


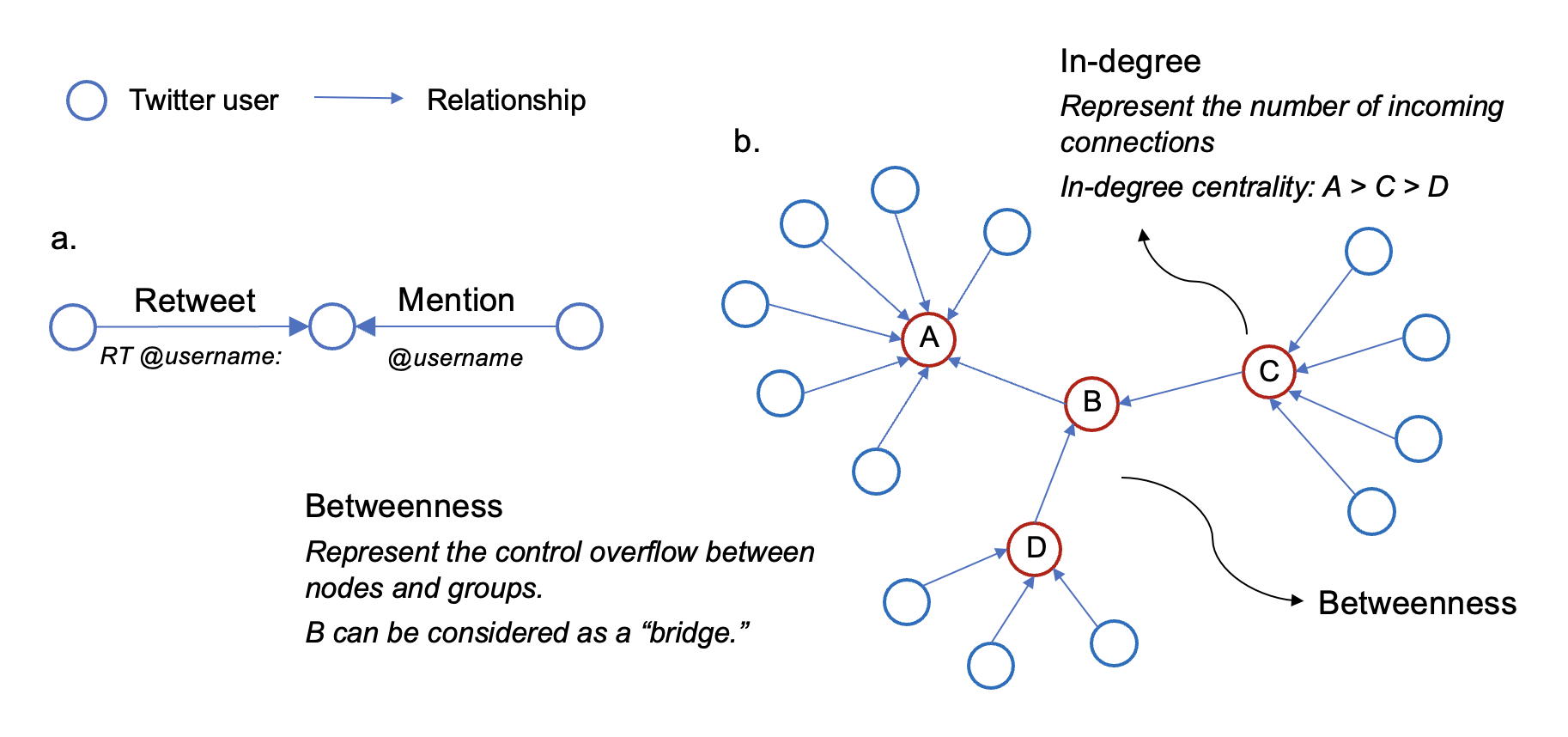


**Figure S1.** Relationships among Twitter users and the degree centrality theory

In addition to the top users based on degree-centrality, we have also investigated the user ranking via betweenness centrality, as attached in the appendix. The top users remain largely the same as using the in-degree centrality: 26 of the top 30 users remain the same in the retweets network. A user with a higher betweenness centrality in the network implies that more information passes through this user. In this context, those top users who communicated toxicity also played a critical role in communicating other toxic information to others. Thus it is sufficient to only use degree-centrality top users for our analysis.

| **Table S1.** Top 30 users based on betweenness centrality in the retweets network. | | | | |
| --- | --- | --- | --- | --- |
| Username | Betweenness | Cluster | Verified | Account type |
| User 1* | 963,382,779.9 | G1 | False | ind_other |
| User 2* | 876,809,948.9 | G2 | False | ind_impact |
| User 3* | 764,206,632 | G4 | True | ind_impact |
| User 4* | 509,923,912.6 | G3 | False | ind_impact |
| User 5* | 352,379,040.6 | G6 | False | ind_impact |
| User 6 | 237,515,582.5 | G2 | False | ind_other |
| User 7* | 232,910,047.8 | G5 | False | ind_impact |
| User 8* | 205,528,733.5 | G7 | False | ind_impact |
| User 9 | 140,960,282.8 | G3 | False | ind_other |
| User 10 | 113,780,200 | G6 | False | ind_other |
| User 11* | 113,734,640.7 | G15 | False | ind_impact |
| User 12* | 98,522,889.35 | G12 | True | ind_impact |
| User 13 | 92,042,632.22 | G4 | False | ind_other |
| User 14* | 90,506,669.18 | G8 | False | ind_impact |
| User 15* | 84,159,899.17 | G9 | False | ind_impact |
| User 16* | 77,754,330.19 | G14 | True | ind_politician |
| User 17 | 73,277,436.47 | G2 | False | ind_other |
| User 18* | 68,551,204.88 | G2 | True | ind_impact |
| User 19* | 67,826,922.9 | G17 | False | ind_impact |
| User 20 | 57,119,996.73 | G7 | False | ind_other |
| User 21* | 51,240,533.17 | G5 | False | ind_impact |
| User 22* | 45,905,200.3 | G23 | False | ind_other |
| User 23* | 45,481,426.53 | G19 | False | ind_impact |
| User 24 | 43,931,889.3 | G6 | False | ind_other |
| User 25 | 43,887,495.78 | G41 | False | ind_other |
| User 26* | 40,839,333.65 | G16 | False | ind_politician |
| User 27* | 39,969,804.56 | G9 | True | ind_impact |
| User 28* | 38,173,697.98 | G10 | False | ind_impact |
| User 29 | 36,740,190.94 | G10 | False | ind_other |
| User 30* | 33,068,415.68 | G20 | False | ind_impact |

*Note: in the table, the symbol “*” indicates that the user also ranked as one of the top 30 users based on the in-degree centrality*.
