# Supplementary material for: Toxicity on Social Media During the 2022 Mpox Public Health Emergency: Quantitative Study of Topical and Network Dynamics
Source: J Med Internet Res. 2024 Dec 12;26:e52997. doi: 10.2196/52997 (PMC11671789; doi:10.2196/52997)
Supplement: Multimedia Appendix 2 [file jmir_v26i1e52997_app2.docx]

## Notes on Topic Modeling

We provide more examples for contextual understanding of the five categories identified through topic modeling and discourse analysis. vowels in inappropriate words are masked.

| **Table S2.** Example topics and tweets of **Disease** | | | |
| --- | --- | --- | --- |
| **Topic** | **Keywords** | **Note** | **Example** |
| 15 | ugly_looks_look_sh*t | Negativity because of physical symptom (disgust) | That MonkeyPox sh*t look so nasty |
| 6 | scary_scared_shit_scaring | Negativity because of mental anxiety (scarcity) | If monkeypox was a person lol I swear that face kills me but no more mate, he's scary as f*ck lol |
| 1 | covid_sh*t_yall_monkeypox | Negativity carried on from related diseases | First Covid now monkeypox? This sh*ts bananas |
| 2 | bullshit_monkeypox_f*ck_monkey | Denial of disease or emergency | i refuse to learn anything about 'monkeypox' f*ck you |

| **Table S3.** Example topics and tweets of **Health Policy and Healthcare** | | | |
| --- | --- | --- | --- |
| **Topic** | **Keywords** | **Note** | **Example** |
| 12 | health_emergency_outbreak_cdc | Declaration and development | Hey CDC, F*ck You and your #monkeypox |
| 10 | vaccine_vax_monkeypox_im | Precaution (vaccine) | Monkeypox vaccine is not the same, you mass media drinking wh*r*. |
| 27 | mask_masks_wear_wearing | Precaution (mask) | People masking up for monkeypox is the ultimate stupidity. |
| 34 | lockdown_lock_sh*t_lockdow | Precaution (lockdown) | As proved lockdowns we're f*ck*n pointless. There was no need for them but cos folk are so gullible; stupid they actually complied without asking questions. Now u know not to comply when there's future lockdowns regarding this Monkeypox which there will. Wake the f*ck up |
| 32 | school_year_kids_senior | Daily life impact (schooling) | people concerned kids are getting monkeypox at school ≠ satanic panic. f*cking idiots co-opting terms to make legitimate concerns look crazy. |
| 36 | gym_crib_going_im | Daily life impact (exercise) | No gym, clubs, or link-ups for awhile for me. This monkeypox sh*t has me shook all over again. |

| **Table S4.** Example topics and tweets of **Homophobia** | | | |
| --- | --- | --- | --- |
| **Topic** | **Keywords** | **Note** | **Example** |
| 7 | sex_anal_transmitted_spread | Medical scapegoating | Monkeypox is very serious, as serious as HIV for gay men having anal sex. The rest of us are Ok. Follow Health Guidelines… avoid anal sex with gay men. Listen to the science! Nuff said? #Canada |
| 16 | ass_eating_butt_asshole | Sexually explicit remarks | Outside eating random ass is insane but eating random ass when monkeypox is a thing is NUTS |

| **Table S5.** Example topics and tweets of **Politics** | | | |
| --- | --- | --- | --- |
| **Topic** | **Keywords** | **Note** | **Example** |
| 11 | biden_ukraine_gates_f*ck | Conspiracy theory | You bet your ass they will.. School shooting, monkeypox. Magically the story has changed away from Biden and his sh*tty gas prices, baby formula shortages, massive inflation, etc |
| 35 | greene_taylor_marjorie_shes | Political remarks | Marjorie Taylor Greene has just humiliated herself once again as she is thick and stupid she doesn't know what the hell she is talking about ok don't listen to her monkeypox is not a sexual transmitted disease there is no evidence of this and kids have not caught it |

| **Table S6.** Example topics and tweets of **Racism** | | | |
| --- | --- | --- | --- |
| **Topic** | **Keywords** | **Note** | **Example** |
| 18 | n*gg*s_nigg*_finna_yall | Racial slur towards African American | Laughing at someone catching monkeypox. You n*gg*s are lame frfr |
| 29 | racist_black_africa_white | Remarks of racism | B*tch I thought monkeypox was some racist shit klan whites were trying to popularize |
